# A Signature of Five Long Non-Coding RNAs for Predicting the Prognosis of Alzheimer's Disease Based on Competing Endogenous RNA Networks

**DOI:** 10.3389/fnagi.2020.598606

**Published:** 2021-01-28

**Authors:** Cai Huaying, Jin Xing, Jin Luya, Ni Linhui, Sun Di, Ding Xianjun

**Affiliations:** ^1^Department of Neurology, Neuroscience Center, School of Medicine, Sir Run Run Shaw Hospital, Zhejiang University, Hangzhou, China; ^2^Department of Orthopedic Surgery, School of Medicine, Sir Run Run Shaw Hospital, Zhejiang University, Hangzhou, China

**Keywords:** bioinformatics, ceRNA, microarray re-annotation, lncRNA, Alzheimer's disease

## Abstract

Long non-coding RNAs (lncRNAs) play important roles in the pathogenesis of Alzheimer's disease (AD). However, the functions and regulatory mechanisms of lncRNA are largely unclear. Herein, we obtained 3,158 lncRNAs by microarray re-annotation. A global network of competing endogenous RNAs (ceRNAs) was developed for AD and normal samples were based on the gene expressions profiles. A total of 255 AD-deficient messenger RNA (mRNA)-lncRNAs were identified by the expression correlation analysis. Genes in the dysregulated ceRNAs were found to be mainly enriched in transcription factors and micro RNAs (miRNAs). Analysis of the disordered miRNA in the lncRNA-mRNA network revealed that 40 pairs of lncRNA shared more than one disordered miRNA. Among them, nine lncRNAs were closely associated with AD, Parkinson's disease, and other neurodegenerative diseases. Of note, five lncRNAs were found to be potential biomarkers for AD. Real-time quantitative reverse transcription PCR (qRT-PCR) assay revealed that PART1 was downregulated, while SNHG14 was upregulated in AD serum samples when compared to normal samples. This study elucidates the role of lncRNAs in the pathogenesis of AD and presents new lncRNAs that can be exploited to design diagnostic and therapeutic agents for AD.

## Introduction

Alzheimer's disease (AD) is the most common cause of dementia and is characterized by age-dependent memory loss and impairment of multiple cognitive functions (Burns and Iliffe, 2009; Braak and Del Trecidi, 2015; Roy et al., 2016). It is the fifth leading cause of mortalities among Americans over 65 years of age (Alzheimer's Association, 2015). Genetic heterogeneity, lifestyle, and environmental factors are crucial to the development of AD. One of the hallmarks of AD pathology is the formation of amyloid β (Aβ) plaques (Hardy and Selkoe, 2002; Mattson, 2004; Karch et al., 2014). However, the pathogenesis of AD in human has not been fully established (Zadori et al., 2018). Many non-coding genes coordinate the development and progression of AD in human (Okazaki et al., 2002; Guttman et al., 2009; Tay et al., 2014). Despite the efforts to design new drugs for AD, there are currently no effective drugs to stop or delay the progression of this disease (Cummings et al., 2017). It is, therefore, imperative to further explore the underlying mechanisms in order to identify potential targets for the development of effective treatments for AD.

MicroRNAs (miRNAs) are small, endogenous, non-coding RNA (ncRNA) molecules that play important regulatory roles in AD (Cheng et al., 2015; Reddy et al., 2017). They bind miRNA response elements (MRE) in the target messenger RNAs (mRNAs). One miRNA may target dozens of mRNA transcripts, whereas one mRNA may contain multiple MREs, which, thus, can be regulated by multiple miRNAs (Bartel, 2009). Studies have shown that specific mRNA transcripts compete for common miRNA binding sites to form competing endogenous RNAs (ceRNAs; Salmena et al., 2011). Therefore, identification of the endogenous competition mechanisms, designing of a framework for prediction, and validation of ceRNAs could reveal new functions of many important transcripts (Thomson and Dinger, 2016).

Several studies have shown that long non-coding RNAs (lncRNAs) are a major type of ncRNAs that can regulate genes at the transcriptional, post-transcriptional, and epigenetic levels (Mercer et al., 2009). Therefore, the imbalance of lncRNA expression is associated with a variety of diseases (Chen et al., 2013), including cancer (Hong et al., 2017) and neurodegenerative diseases (Riva et al., 2016). It has been documented that knockdown of lncRNA NEAT1 in the AD cell model attenuated the degree of Aβ-induced inhibition of the extraction, promoted myocardial cell apoptosis, and weakened the p-tau level (Ke et al., 2019; Zhao et al., 2019). However, little is known about the role of lncRNAs in AD. Theoretical and experimental studies have shown that a large number of miRNA-binding sites exist on different types of RNA transcripts, suggesting that different RNA transcripts with miRNA-binding sites can regulate each other by competing for shared miRNAs, thus becoming ceRNAs; (Tay et al., 2014). Importantly, lncRNAs competing with miRNA target mRNA for miRNA molecules, thereby, regulating miRNA-mediated target repression (Tay et al., 2014). Wang et al. (2018) used microarray analysis to identify circRNA-related ceRNA networks in the hippocampus of Aβ1-42 induced AD rat models. Elsewhere, Cai et al. (2017) found that Rpph1 upregulates the expression of CDC42 and promotes the formation of dendritic spine in hippocampal neurons by competing with miR-330-5p (Cai et al., 2017). It was reported that ciRS-7 acts as a competitive endogenous miRNA sponge that inhibits the function of miRNA-7 in AD-affected brains (Lukiw, 2013). However, the mechanisms by which ceRNA associates with AD have not been established.

In this study, we obtained the expression profile data of 629 patients with AD and control samples from eight datasets in the gene expression omnibus (GEO) database. The mRNA, miRNA, and lncRNA expression profiles were obtained through chip re-annotation, data integration, and standardization. The miRNA-mRNA and miRNA-lncRNA interaction data were obtained from starBase database. The hypergeometric distribution approach was used to establish a background ceRNA regulation network. Expression correlation was used for pruning after which functional module mining was performed through a co-expression network to identify candidate diagnostic biomarkers or potential therapeutic targets.

## Materials and Methods

### Data Collection and Processing

We searched the GEO database and the Affymetrix Human Genome U133 Plus 2.0 Array chip platform to identify gene expression profile data using the keyword “Alzheimer's disease.” Samples sizes with ≥30 datasets and 7 datasets were obtained. In addition, the dataset GSE16759, which simultaneously detected the expression profiles of miRNA and mRNA, included eight sets of 629 samples. Forty samples of undefined AD were excluded, and finally 589 samples were included as shown in Table 1. For the expression profile data, the raw expression level data (.CEL files), for each sample was downloaded and normalized using the robust multi-array (RMA) of the R package affy (Gautier et al., 2004). Next, the batch effect was removed using the combat of the R package SVA (Leek et al., 2012). The microarray data were derived from the GEO (accession GSE133349). We matched the probes with their corresponding genes. In cases where multiple probes corresponded to one gene, the median was expressed. Where one probe corresponded to multiple genes, such a probe was excluded.

**Table 1:** Incorporated gene expression omnibus (GEO) dataset information.

	**No. of Samples**	**No. of AD**	**No. of Normal**	**No. of removed**	**Matched miRNA**
GSE16759	8	4	4	0	Yes
GSE28146	30	22	8	0	NO
GSE48350	253	80	173	0	NO
GSE5281	161	87	74	0	NO
GSE53890	41	0	41	0	NO
GSE84422	102	34	28	40	NO
GSE9770	34	34	0	0	NO

### Re-Annotation of Expression Data

To obtain the latest lncRNA expression profile, lncRNA re-annotation was performed using the HG-U133 2.0 array. First, the GENCODE Release 21 version of lncRNA transcript sequences was downloaded from GENCODE. Then, probe clusters sequences were aligned to lncRNA sequences using seqmap (Hawkins et al., 2011), and mismatch was set to 0. At least 11 clusters were selected and compared to probes on the same lncRNA to confirm the success of re-annotation. Finally, 3,943 Affymetrix probe sets (3,158 lncRNA) were included in subsequent analyses.

### miRNA Interaction Data

StarBase V3.0 database (Li et al., 2014) is designed to integrate large-scale CLIP-Seq (HITS-CLIP, PAR-CLIP, iCLIP, and CLASH) data to decode interactive networks. Five predictive algorithms were used to predict miRNA target genes, including TargetScan, miRanda, Pictar, PITA, and RNA22. Data of 1,055,319 miRNA-mRNA interactions for 484 miRNAs and 15,064 mRNAs and of 63,698 miRNA-lncRNA interactions for 642 miRNAs and 3,789 lncRNAs were downloaded from the starBase V3.0 database.

### Construction of ceRNA Network

As described by Fan et al. (2018), the hypergeometric model was used to construct a ceRNA network. In summary, the miRNAs shared by mRNA and lncRNA were used to calculate the interactive probability of mRNA-lncRNA networks. The threshold was FDR < 0.05. When more than three miRNAs were found to be shared between mRNA and lncRNA, they were selected. The value of *p* was calculated using the following formula:

(1) $$\begin{array}{l} p\;value=1-\overset{r-1}{\underset{k=0}{\sum }}\frac{\left(\begin{array}{c} t\\ k \end{array}\right)\left(\begin{array}{c} m-t\\ n-k \end{array}\right)}{\left(\begin{array}{c} m\\ n \end{array}\right)} \end{array}$$

where “m” represents the total number of miRNAs in starBase database, “t” represents the number of miRNAs that interacted with the mRNA, “n” represents the number of miRNAs that interacted with the lncRNA, and “r” represents the number of miRNAs shared between mRNA and lncRNA.

Correlations among mRNA-lncRNA pairs were calculated in the expression profiles of normal and AD samples, and the correlation coefficient > 0.25 and FDR < 0.05 were selected to identify the ceRNA global network.

### Identification of Dysregulated ceRNA Networks

We compared the global networks of ceRNAs in normal and AD samples and selected the intersection of two networks. Furthermore, the difference between the Pearson's correlation coefficients of mRNA-lncRNA in two ceRNA networks was calculated. With 1,000 random readings as the statistical background, the probability (*p* ≤ 0.05) of Pearson's correlation coefficient difference was applied to identify dysregulated mRNA-lncRNA.

### Identification of KEGG Pathways Associated With lncRNA

Each sample was subjected to the Kyoto Encyclopedia of Genes and Genomes (KEGG) pathway analysis of ssGSEA using the R package GSVA38 to identify pathways associated with lncRNA expression, applying a correlation > 0.5.

### Data and Code

To facilitate the reproduction of our results, the eight datasets were uploaded to the GEO database (https://www.ncbi.nlm.nih.gov/geo/query/acc.cgi), with the ID: GSE133349.

### Real-Time Quantitative Reverse Transcription PCR

TRIzol reagent (ThermoFisher Scientific, 15596026) was used to extract total RNA from serum samples. The synthesis of total RNA into cDNA was performed according to the instructions of the Reverse Transcription Kit (TshermoFisher Scientific, BTK1622). The ABI 7500 real-time PCR instrument was used to perform the real-time PCR reaction using the quant one-step real-time quantitative reverse transcription PCR (qRT-PCR) Kit [FP303, Tiangen Biochemical Technology (Beijing) Co., Ltd.]. The primers used were as follows: SNHG14 forward, ATGAGCTGACAACCTACTCC and reverse, AAGTCATCTTCTGCAAGGGT; PART1 forward, CCCTTTCACTATGAAGGACC and reverse, ATTTACCCGTCCAGTTCTG; NNT-AS1 forward, AGAAACAGGTCTAAAGACCCT and reverse, TTCTTGGCATCTCTGAGCA; AC093010.3 forward, TGATGTGTTTGCTATCTGCT and reverse, TGTTAACAGCTAGCCATTCAG; ARMCX5-GPRASP2 forward, AAGAGAAGGGATAGAGTGGTG and reverse, CTTCTGTCATAGAAATTTCCCTCTC; GAPDH forward, TCAAGATCATCAGCAATGCC and reverse, CGATACCAAAGTTGTCATGGA; GAPDH were used as internal controls. 2^−ΔΔCT^ was used to calculate the relative expression.

### Statistical Analysis

Statistical analyses were performed using R 3.4.3, and all analyses (non-specific description) were performed with default parameters. Group comparisons were performed using the Student's *t*-test, correlations were determined using Pearson's correlation coefficient, g:profiler (Reimand et al., 2019) was used for gene enrichment analysis, and Cytoscape (Shannon et al., 2003) (http://www.cytoscape.org/) was used for network visualization.

## Results

### ceRNA Network of miRNA-mRNA in AD

**Figure 1:**
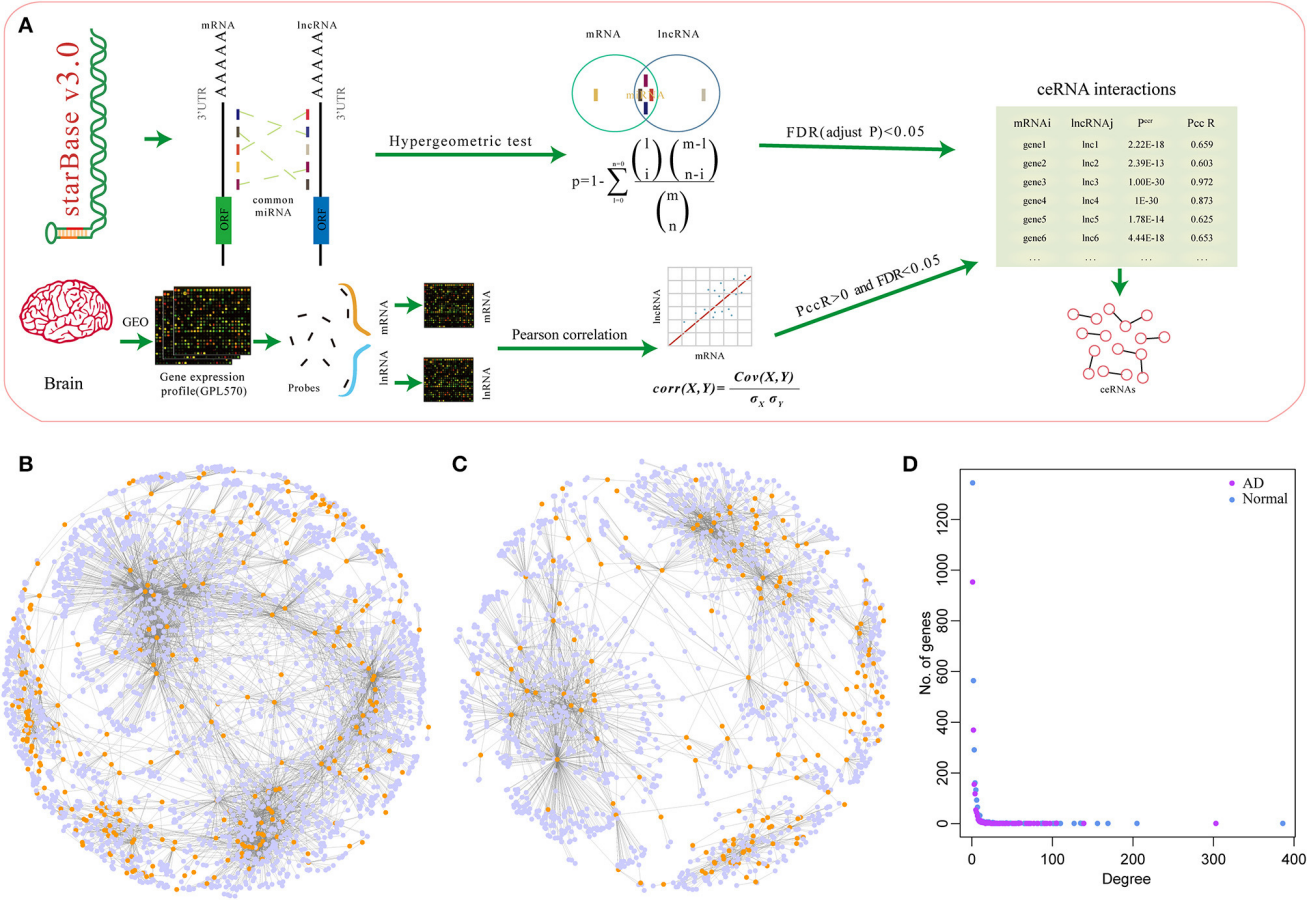
MicroRNA (miRNA)-mediated competing endogenous RNA (ceRNA) network associated with mRNA in Alzheimer's disease (AD). **(A)** The construction process of the ceRNA network. **(B)** The ceRNA network in normal samples, the light blue nodes in the figure represent genes and the dark yellow nodes represent long non-coding RNA (lncRNA). **(C)** The ceRNA network in AD samples, the light blue nodes in the figure represent genes and the dark yellow nodes represent lncRNA. **(D)** The degree distribution of ceRNA networks in normal and AD samples, the horizontal axis represents the degree of network nodes, the vertical axis represents the number of genes, the blue dots represent the degree distribution in normal samples, and the purple dots represent the degree distribution in AD samples.

To investigate the potential role of miRNA-mediated ceRNA networks in AD, we analyzed the ceRNA landscape in normal samples and AD samples based on the expression profiles (Figure 1A). Using the hypergeometry test, we identified 164,494 miRNA-mediated mRNA-lncRNA pairs with 7,484 genes. To construct a sample-specific ceRNA network, the correlation of these mRNA-lncRNA pairs in the expression profiles of normal and AD samples was determined. The competitive endogenous RNAs (ceRNAs) network was established using the correlation coefficient > 0.25 and FDR < 0.05. A total of 2,571 mRNA-lncRNA pairs and of 255 lncRNAs for 2,551 genes were identified in normal samples (Figure 1B), while 3,353 mRNA-lncRNA pairs and of 227 lncRNAs for 1,619 genes were identified in AD samples (Figure 1C). The two ceRNA network distributions exhibited a consistent law distribution (Figure 1D) and were consistent with biological network characteristics, indicating that ceRNA plays an important role in AD that is similar to other biological regulatory networks.

### Co-expression Characteristics of mRNA-lncRNA in ceRNA Network and Dysfunctional ceRNA Network

**Figure 2:**
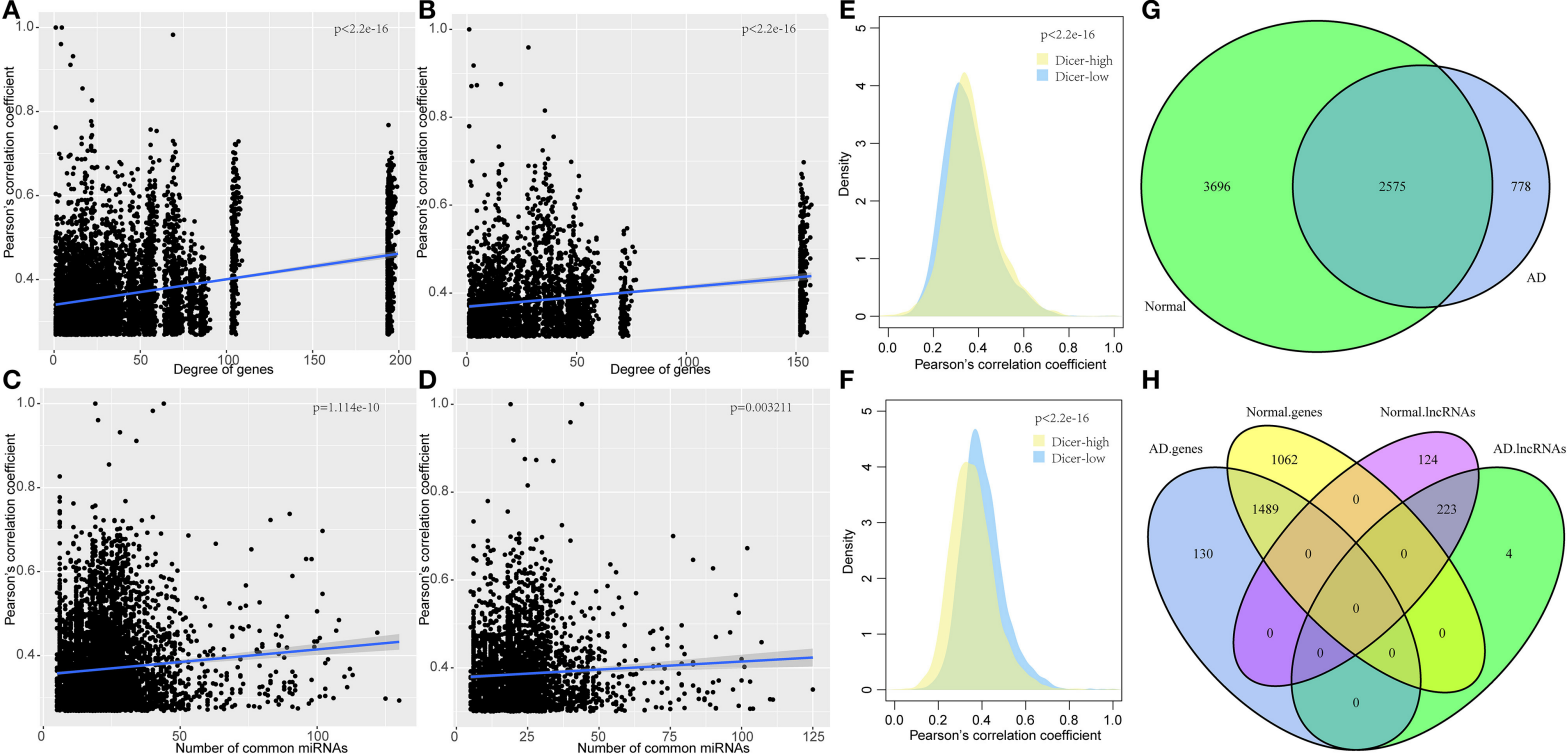
Co-expression characteristics of mRNA-lncRNA in ceRNA network and dysfunctional ceRNA network. **(A)** Degree of correlation between the ceRNA network and co-expression of mRNA-lncRNA in normal samples, the horizontal axis represents the degree of the gene and the vertical axis represents the correlation coefficient. **(B)** Degree of correlation between ceRNA network and co-expression of mRNA-lncRNA in AD samples, the horizontal axis represents the degree of the gene and the vertical axis represents the correlation coefficient. **(C)** Correlation between the number of miRNA shared by mRNA-lncRNA and the co-expression of mRNA-lncRNA in ceRNA networks in AD samples, the horizontal axis represents the number of miRNA and the vertical axis represents the correlation coefficient. **(D)** Correlation between the number of miRNAs shared by mRNA-lncRNA and the co-expression of mRNA-lncRNA in ceRNA networks in AD samples, the horizontal axis represents the number of miRNA and the vertical axis represents the correlation coefficient. **(E)** Co-expression pattern of Dicer-low and Dicer-high groups in normal samples, the vertical axis represents the correlation coefficient. **(F)** Co-expression pattern of Dicer-low and Dicer-high groups in AD samples, the vertical axis represents the correlation coefficient. **(G)** Venn diagram of the intersection of ceRNA networks in normal samples and AD samples. **(H)** Intersection of genes and lncRNA in ceRNA networks in normal and AD samples.

To explore the co-expression relationship of the mRNA-lncRNAs in the ceRNA network, we analyzed the relationship between network degree and mRNA-lncRNA co-expression. The network degree was significantly positively correlated with mRNA-lncRNA co-expression (Figures 2A,B). Genes with a high network degree showed higher co-expression relationships, and many miRNAs were found to be shared by mRNA and lncRNA. A high correlation coefficient was found between the corresponding mRNA and lncRNA (Figures 2C,D). Studies have shown that the expression levels of miRNA determine the activity of ceRNA networks (Ala et al., 2013). We, therefore, studied the effect of miRNA expression on ceRNA networks in AD. Dicer is a key enzyme that regulates miRNA processing and maturation. Herein, samples were divided into two groups based on the expression level of Dicer: Dicer-low and Dicer-high. In normal samples, the co-expression pattern in the Dicer-high group was higher than that in the Dicer-low group, while opposite results were obtained in AD samples (Figures 2E,F), suggesting that moderate expression of miRNAs in AD is necessary to maintain the ceRNA network, but high expression of miRNAs may lead to degradation of many genes.

Comparison of the ceRNA networks in normal and AD samples showed that the number of ceRNAs in AD is only 53.5% to that of normal samples, suggesting a large number of ceRNA regulation abnormalities in AD samples, compared to normal samples. Notably, 2,575 (41.1%) ceRNAs were unchanged while 3,696 (58.9%) ceRNAs disappeared from the AD samples (Figure 2G). Analysis of genes and lncRNAs showed that 1,489 (58.4%) genes were shared between the two networks while 1,062 (41.6%) genes disappeared from the ceRNA network of AD (Figure 2H). These findings imply a high dysregulation of ceRNA in AD. Furthermore, we randomly divided the samples into two queues, the training queue and the verification queue, to determine the dysregulation of ceRNA networks. In the training queue, Pearson's correlation coefficients of mRNAs in two ceRNA networks were calculated, and those with *p* ≤ 0.05 were selected as the background of the random network. A total of 294 dysregulated mRNAs-lncRNAs were identified in training queue, and a total of 333 dysregulated mRNA-lncRNAs were identified in the verification cohort by the same method. In addition, 258 dysregulated mRNA-lncRNAs were selected from all samples by the same method. By comparing the dysregulated mRNA-lncRNAs in the three cohorts, a high degree of overlap was found between them (Supplementary Figure 1A). This showed the stability of dysregulated mRNA-lncRNAs in different samples. Finally, in all samples, we identified 255 dysregulated mRNA-lncRNAs. Among them, 103 pairs were downregulated in AD, including 96 genes and 30 lncRNAs, while 152 pairs were upregulated in AD, including 146 genes and 53 lncRNAs (Supplementary Figure 1B). Notably, 71 lncRNAs in the statistical network were co-expressed with other mRNAs, with an average proportion of the same type accounting for 95.1%. The co-expression of lncRNA and other mRNAs tended to be the same type of aggregation, suggesting that lncRNA expression levels play a key role in the dysregulation of ceRNA networks in AD.

### Functional Enrichment Analysis of the Dysregulated ceRNA Networks in AD

**Figure 3:**
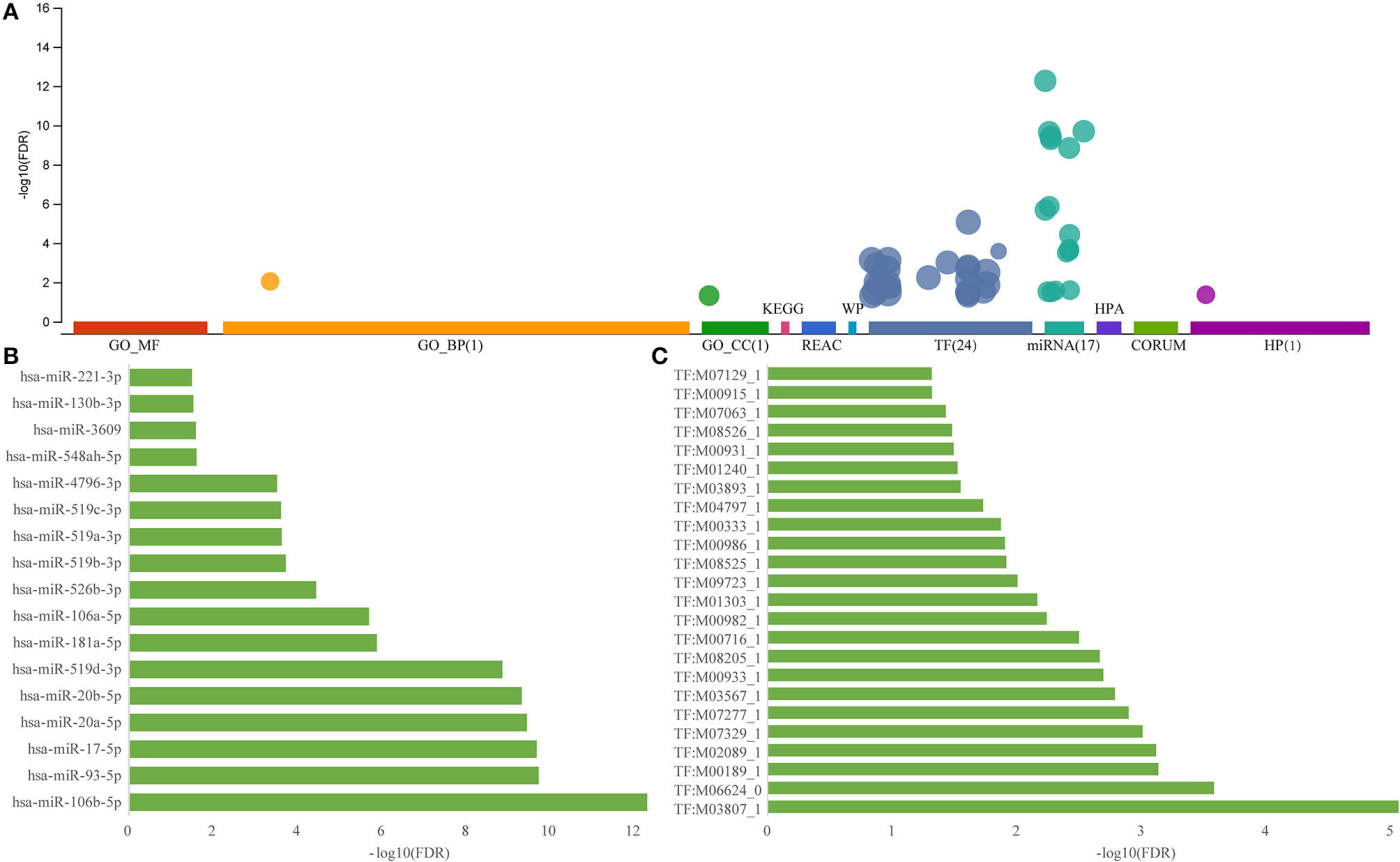
Functional enrichment analysis of dysregulated ceRNA network in AD. **(A)** Enrichment analysis for the 231 genes in ceRNA network, the horizontal axis represents different databases and the vertical axis represents enrichment significance FDR. **(B)** miRNA enriched by 231 genes in the ceRNA network, the horizontal axis represents enriched significance FDR and the vertical axis represents miRNA. **(C)** Transcription factors enriched from 231 genes of the ceRNA network, the horizontal axis represents enriched significance FDR and the vertical axis represents transcription factors.

To elucidate on the functional implications of the ceRNA network, functional enrichment analysis was performed on all 231 genes in the ceRNA network. Results showed that these genes were enriched in two gene ontology (GO) terms and were not associated with any pathway (Figure 3A), implying that the dysfunctional ceRNA network lacked specific functions to be considered a large module. These genes were enriched in 24 transcription factors and 17 miRNAs, indicating that they were highly shared between transcription factors and miRNAs. The most significantly enriched miRNA, hsa-miR-106b-5p, was found to be associated with AD susceptibility and is a potential peripheral blood biomarker for AD (Yilmaz et al., 2016; Figure 3B). The most significantly enriched transcription factor, M038071 (SP2), is an important marker in cancer chemotherapy (Vizcaino et al., 2015; Figure 3C). Thus, ceRNA is used as a small module in AD instead of a single or large module.

### Identification of Potentially Dysregulated miRNA-Target Pairs in the Dysregulated ceRNA Network

**Figure 4:**
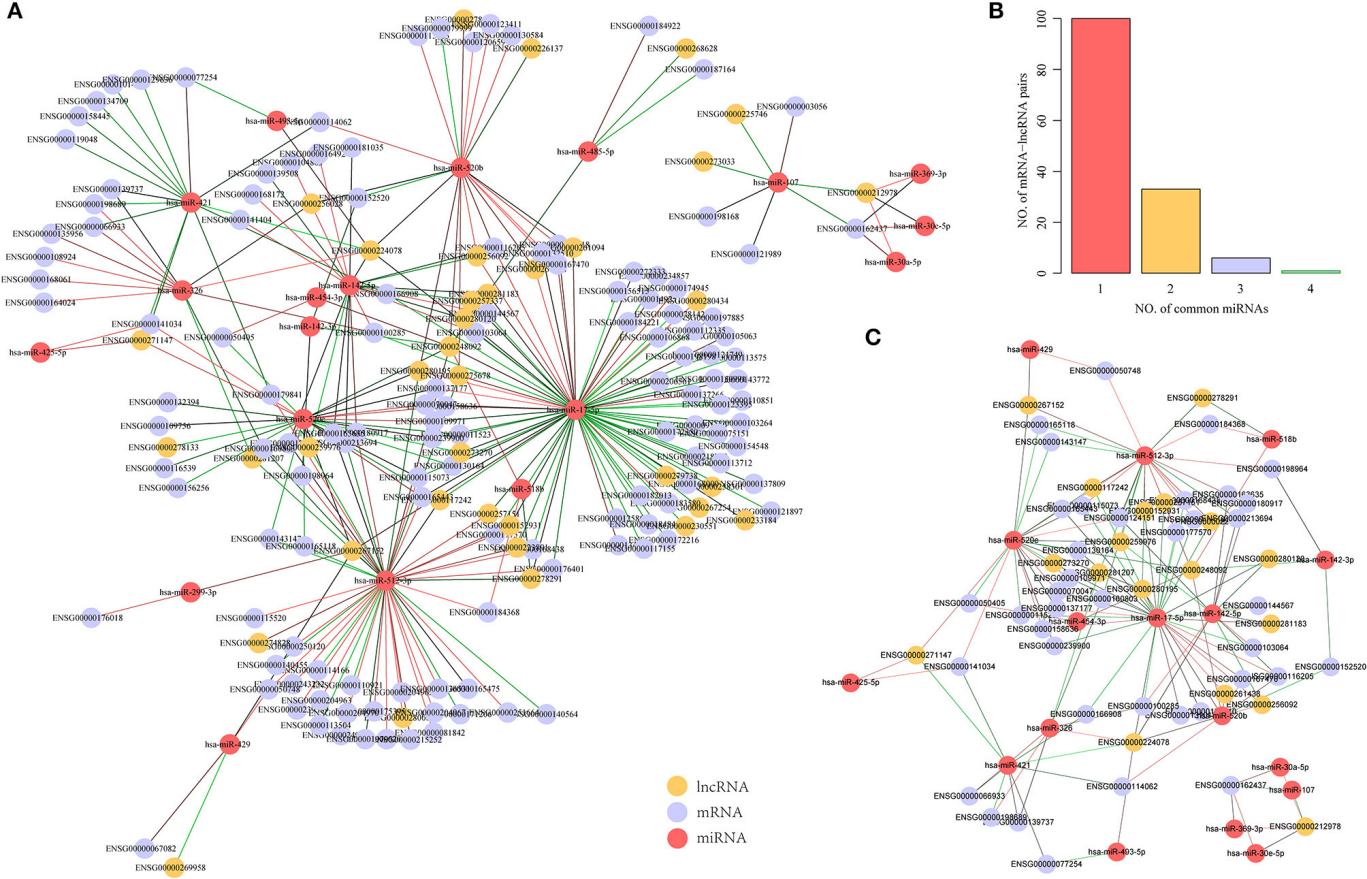
Identification of potential dysregulated miRNA-target pairs in a dysregulated ceRNA network. **(A)** Dysregulated mRNA-miRNA-lncRNA ternary relationship network in AD samples, the green line indicates downregulated genes in AD samples while the red line indicates upregulated genes in AD samples, the yellow dot represents lncRNA, the blue dot represents mRNA, and the red dot represents miRNA. **(B)** mRNA-lncRNA-shared dysregulated miRNAs number distribution, the horizontal axis represents the number of miRNA and the vertical axis represents the number of mRNA-lncRNA pairs. **(C)** mRNA-lncRNA shared dysregulated miRNA number >1 mRNA-miRNA-lncRNA ternary relationship network, the green line indicates downregulation in AD samples, the red line indicates upregulation in AD samples, the yellow dots represent lncRNA, the blue dots represent mRNA, and the red dots represent miRNA.

We further used the paired samples of miRNA, mRNA, and lncRNA expression profiles from the GSE16759 dataset to identify dysregulated mRNA-miRNA and miRNA-lncRNA pairs in AD samples. Using *p* < 0.05 as the cut-off value for selection, 86 pairs of miRNA-lncRNA and 111 pairs of mRNA-miRNA, including 19 miRNAs, 131 genes, and 37 lncRNAs, were identified. Interestingly, unlike mRNA-lncRNA, these dysregulated miRNA-target pairs were downregulated in AD samples (Figure 4A), and this phenomenon is consistent with negative regulation for miRNA and mRNA. The number of miRNAs shared by mRNA-lncRNA in the ternary network revealed that 71.4% of mRNA-lncRNA pairs contain only one dysregulated miRNA (Figure 4B). We constructed a ternary interaction network in which more than one mRNA-lncRNA pair of dysregulated miRNAs was shared for 40 pairs of mRNA-lncRNA, which contained 39 genes, 18 lncRNAs, and 17 miRNAs (Figure 4C). Notably, three of the four miRNAs in ENSG00000162437 (RAVER2) and ENSG00000212978 (LOC339803) pairs that shared the highest number of dysregulated miRNAs were significantly downregulated in AD (*p* < 0.05; Supplementary Figure 2), indicating that the shared dysregulated miRNAs were more likely to be related to ceRNAs of mRNA-lncRNA.

### Identification of AD-Related lncRNAs Based on ceRNAs

**Figure 5:**
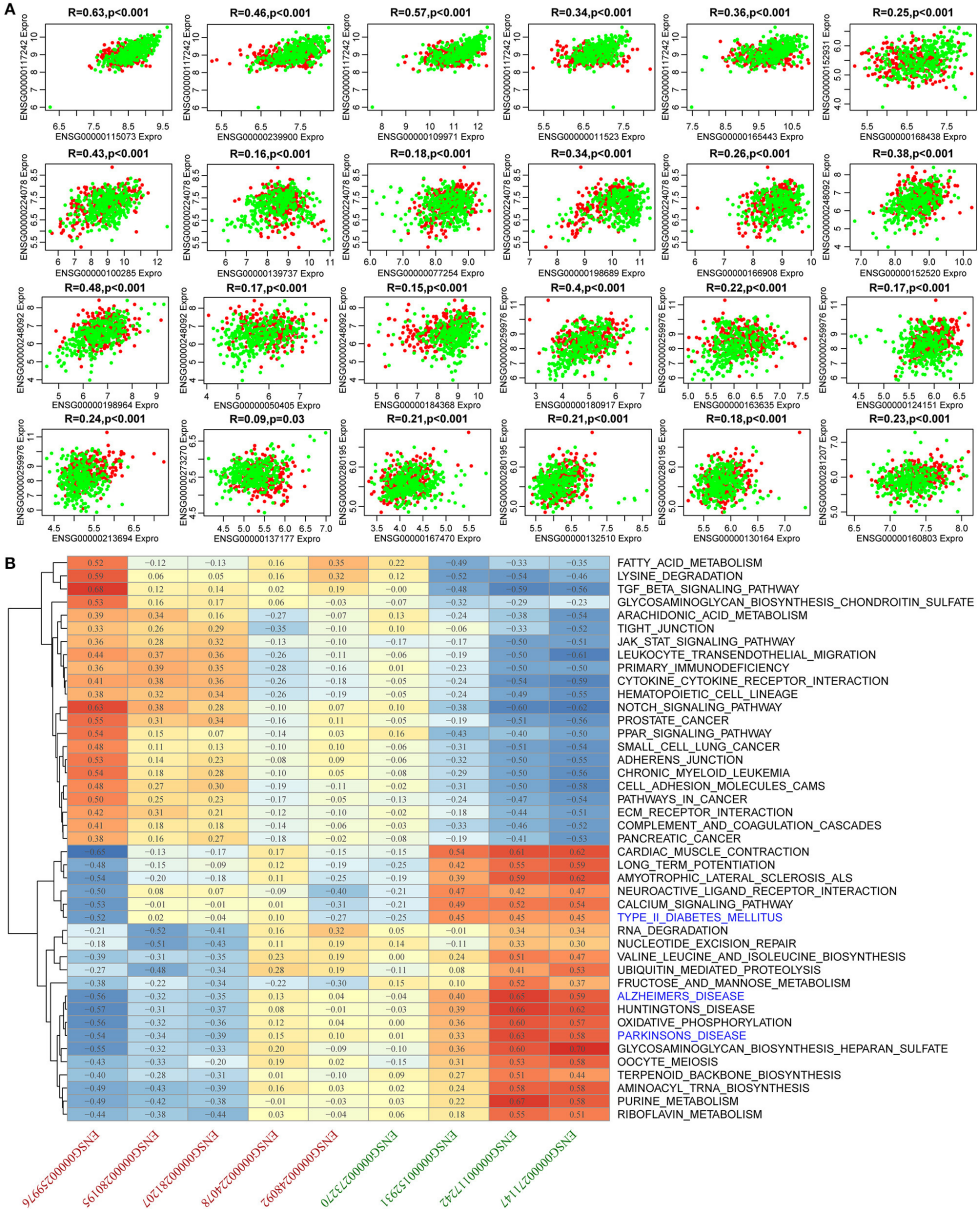
Expression and functional analysis for 24 pairs of simultaneous differences of mRNA and lncRNA. **(A)** Correlation among 24 pairs of mRNA-lncRNA expression. The horizontal axis shows the genes, the vertical axis represents lncRNAs, the green represents normal samples, and the red represents AD samples. **(B)** Pearson's correlation clustering heat map of nine lncRNA expressions and KEGG pathway enrichment scores. The red axis represents lncRNAs that were upregulated in AD, and the green axis represents the lncRNAs that were downregulated in AD.

To identify AD-related lncRNAs, we analyzed the differential expression of 18 lncRNAs in AD from more than one mRNA-lncRNA pair sharing dysregulated miRNAs. We found nine (52.9%) lncRNAs to be significantly differentially expressed (Supplementary Figure 3). Similarly, we examined the differential expression of 39 genes in AD and found that 32 (82.1%) genes were significantly differentially expressed (Supplementary Figure 4), suggesting that the constructed dysregulated ceRNA network could effectively screen for disease markers. There were 24 differential pairs with simultaneous differential differences of mRNA and lncRNA. These 24 pairs had significant correlations. Among them, the average correlation coefficients of ENSG00000117242 and ENSG00000224078 and their corresponding five genes ranged between 0.48 and 0.27, while the average correlation coefficient of ENSG00000248092 and ENSG00000259976 and their respective four genes was between 0.3 and 0.28 (Figure 5A). In summary, the lncRNA-mRNA pairs that were found to be dysregulated in AD were significantly co-expressed, while 9 lncRNAs and 24 mRNAs may be potential biomarkers in AD. Most of these 24 genes have been reported to be associated with AD, such as low-density lipoprotein receptor (LDLR) overexpression which increases β-amyloid clearance and decreases amyloid deposition (Krishnan et al., 2020), HSPA8 is downregulated in multiple brain regions in AD (Silva et al., 2014), and SLC9A6 mutation causes intellectual disability (Garbern et al., 2010). These nine lncRNAs have not been reported to be associated with AD, and based on this, we further analyzed them. Moreover, KEGG pathway analysis was performed on the samples using ssGSEA to determine the pathways associated with the nine lncRNAs. KEGG pathways with a correlation exceeding 0.5 were selected and enriched in 43 pathways. These pathways were divided into two categories, one of which is positively correlated with the highly expressed lncRNAs in AD, the other category is positively related to lncRNAs, which are lowly expressed in AD. Pathways associated with downregulated lncRNAs were mainly those related to senile disease-related pathways and tumor, as well as immune-related pathways. These pathways include AD, Parkinson's disease, and type II diabetes mellitus (Figure 5B). These pathways play important roles in the development of AD, and the nine lncRNAs regulate the pathogenesis of AD.

### Diagnostic Power of Dysregulated lncRNAs in AD

**Figure 6:**
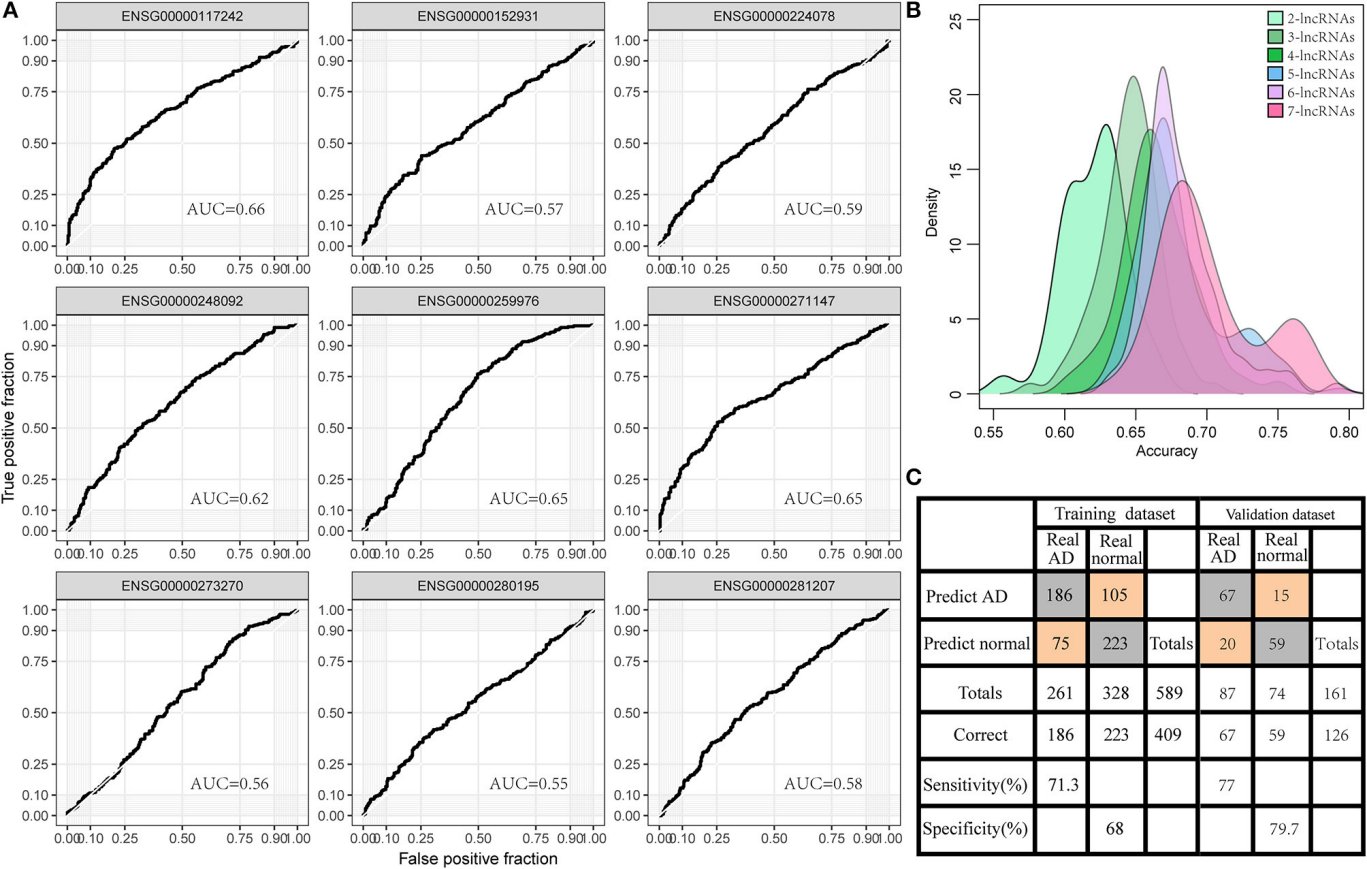
Diagnostic value of lncRNA in AD. **(A)** The area under the curve (AUC) of nine gene expression profiles for classification of AD samples. **(B)** The accuracy distribution of different number of lncRNA combinations among 502 combinations of nine genes for predicting AD. **(C)** The predicted classification models of the signature of five lncRNAs in the training set and the validation set.

**Figure 7:**
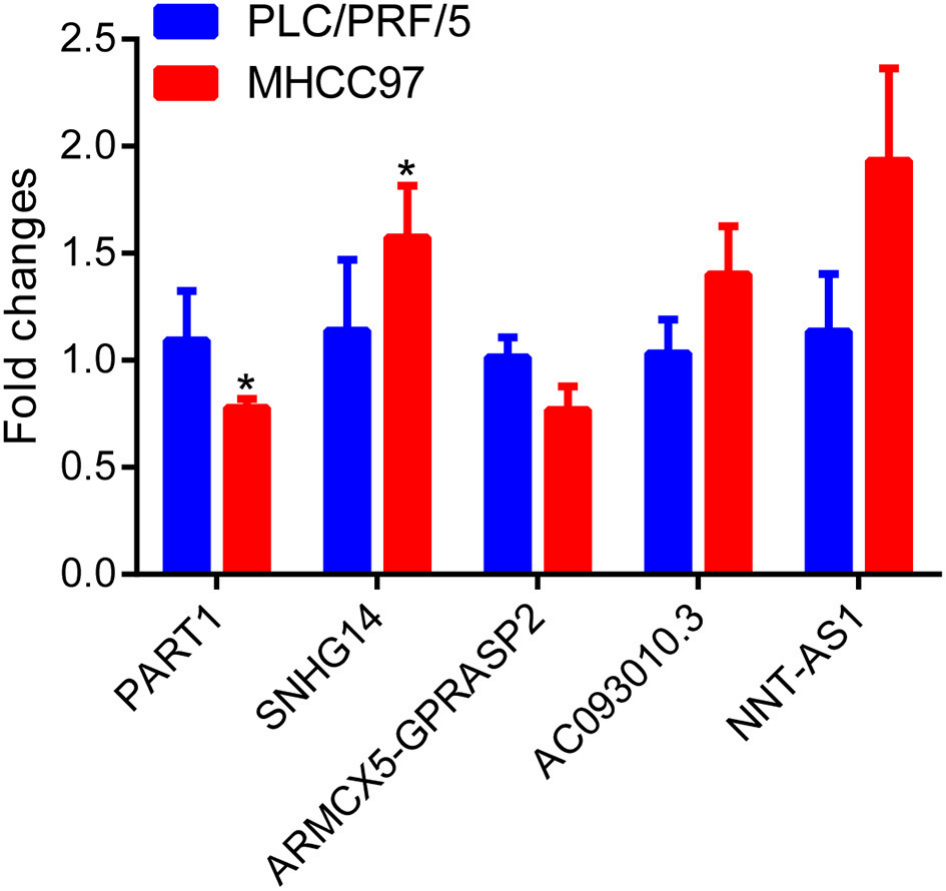
Differential expression analysis of five lncRNAs in AD patient serum using real-time quantitative reverse transcription PCR (qRT-PCR) assay.

To investigate the diagnostic value of lncRNA in AD, a receiver operating characteristic (ROC) curve classification was performed for the nine lncRNAs in AD samples and normal samples. Results showed that each of the nine lncRNAs could diagnose AD with an average area under the curve (AUC) of 0.6 (Figure 6A). However, the diagnostic value of single molecules may be limited. Thus, we randomly combined nine genes to obtain 502 combinations and used these combined expression profiles to establish support vector machine (SVM) models for the diagnosis of AD. We found that the accuracy of the lncRNA combinations gradually increased as the number of lncRNAs increased (Figure 6B). The combination with the highest accuracy contained five lncRNAs: ENSG00000224078 (SNHG14), ENSG00000152931 (PART1), ENSG00000248092 (NNT-AS1), ENSG00000259976 (AC093010.3), and ENSG00000271147 (ARMCX5-GPRASP2). The SVM was used to establish a diagnostic model, which was tested using a 10-fold cross-validation method. This test showed that the classification accuracy of the diagnostic model was 69%; 409 of the 589 samples were correctly classified. The sensitivity of the diagnostic model for AD was 71.3% while its specificity was 68%. We further used the GSE5281 dataset to verify the accuracy of the diagnostic model, with results showing a classification accuracy rate of 78.3%; 126 of the 161 samples were correctly classified. The sensitivity of the model for AD was 77% while the specificity was 79.7% (Figure 6C). These results indicate that the diagnostic model could effectively distinguish between patients with AD and normal control populations. Five lncRNAs showed a good potential to be reliable biomarkers for the diagnosis of AD. To confirm the role of five lncRNAs, qRT-PCR was used to detect the expression levels of lncRNA in the serum samples of patients with AD. PART1 and ARMCX5-GPRASP2 expressions were found to have similar trend expressions with bioinformatics analysis (Supplementary Figure 3). However, only PART1 was downregulated while SNHG14 was upregulated in the serum samples of patients with AD when compared to normal samples (Figure 7).

## Discussion

Advances in high-throughput experimental techniques have enabled a comprehensive analysis of ceRNA interactions using the gene expression correlation approach. In addition, public databases and computational methods such as starBase provide platforms for the analysis of CLIP-seq-supported miRNA-mRNA interactions (Li et al., 2014), thereby promoting ceRNA research. Previous studies have explored functional lncRNAs involved in AD using ceRNA and lncRNA-mRNA networks (Wang et al., 2017). A novel computational approach has recently been proposed to identify sponge interactions by integrating gene co-expression information in breast cancer (Paci et al., 2014). Research on gene co-expressions in the ceRNA network has mainly focused on cancer (Shao et al., 2015; Li et al., 2018; Gao et al., 2019). Unlike AD, for which no database system has been established to store gene expression data generated from studies in the AD field, cancer datasets are available from The Cancer Genome Atlas (TCGA) database. This limits research into the pathogenesis and progression of AD. In this study, we obtained the gene expression data of AD from the GEO database, which stores a large amount of mRNA and miRNA-related expression profiles. Compared to the TCGA database, GEO's data are decentralized; therefore, can only be retrieved manually. A total of 589 samples and 3,943 lncRNA probes were obtained by microarray re-annotation to study the ceRNA regulatory mechanism of miRNA-mediated mRNA-lncRNA in AD. Given the large sample size and numerous lncRNAs in AD that were analyzed in this study, our results are reliable (Mendoza et al., 2000).

Competitive endogenous RNA regulatory networks comprising mRNA, miRNA, and lncRNA play important roles in the pathogenesis of human diseases (Zhao et al., 2016; Chen et al., 2017). The ceRNA network provides a platform for studying interactions between lncRNAs and mRNAs. For example, in an AD mouse model, the lncRNA-associated ceRNA networks were found to be mainly involved in synaptic plasticity as well as memory (Akap5), and the regulation of Aβ-induced neuroinflammation (Klf4; Ma et al., 2020). By analyzing an AD neurofibrillary tangles (NFTs) lncRNA-mRNA network (NFTLMN), three lncRNAs (AP000265.1, KB-1460A1.5, and RP11-145M9.4) which are highly associated with AD NFTs were identified (Wang et al., 2017). In this study, we calculated the Pearson's correlation coefficients of all lncRNA-mRNA interactions under normal and disease states. We found that abnormal interactions of ceRNA function as a “switch” that modulates the pathogenesis of AD. Systematic evaluation of the dysregulated ceRNAs in AD revealed that mRNAs were enriched in multiple transcription factors and miRNAs, which shared regulatory patterns during gene expression.

In the past, the molecular mechanisms of the pathogenesis of AD were mainly studied with a greater focus on coding genes and miRNAs. Studies have shown that the aberrant expression of lncRNA is closely associated with the occurrence and development of many diseases (Khorkova et al., 2015). Indeed, some lncRNAs have been found to be therapeutic targets for the clinical treatment AD. Accumulating evidence indicates that miRNA-lncRNA interactions play important roles as ceRNAs (Zhou et al., 2016). lncRNAs, LINC00836, and DCTN1-AS1 potentially contribute to AD immune-related phenomena by regulating AD-related immune genes (Xu and Jia, 2020). Knockdown of lncRNA SNHG1 was found to exhibit neuronal protective effects through the repression of KRENEN1 by acting as a ceRNA for miR-137 in an *in vitro* cell model of AD (Wang et al., 2019). In AD, lncRNA-related ceRNA networks have been found to regulate its development. However, none have systematically analyzed the function of lncRNA-related ceRNA networks in AD. In this study, we identified ceRNAs that were deregulated in AD by analyzing mRNA, miRNA, and lncRNA expression profiles. As a result, nine key lncRNAs were identified. Functional analysis revealed that these dysregulated lncRNAs were highly correlated with aged dementia and Parkinson's disease. In terms of the diagnostic ability of the lncRNAs, we found that the diagnostic accuracy of the lncRNA combinations increased as the number of lncRNA in the combination increased. Specifically, five lncRNAs, ENSG00000224078 (SNHG14), ENSG00000152931 (PART1), ENSG00000248092 (NNT-AS1), ENSG00000259976 (AC093010.3), and ENSG00000271147 (ARMCX5-GPRASP2), were found to have a good diagnostic ability for AD. qRT-PCR results also revealed that these lncRNAs were differentially expressed in serum samples of patients with AD.

Although these results are based on bioinformatics analysis, they elucidate the occurrence and development of AD. However, we acknowledge some limitations in this study. First, we used probe re-annotation pipelines to identify AD-related functions of lncRNA, which are widely used by many bioinformatics studies. However, this pipeline filters out many lncRNA that do not match the probe sequence. Second, we integrated gene expression and miRNA-target interactions to identify dysregulated ceRNA interactions, and our results would be more reliable if a more precise method was used. Third, this work, as a study in the field of bioinformatics, was often aimed at verifying the accuracy and reliability of the lncRNA-mRNA network and lncRNA-related functional modules or the diagnostic potential of the biomarker lncRNA by statistical significance and scientific literature verification. In future studies, we plan to conduct well-designed experiments to explore detailed mechanisms in order to verify the results presented in this study.

Moreover, we utilized a large sample cohort to screen for ceRNA networks that were dysregulated in AD using the lncRNA re-annotation system. We showed that this novel method is effective for studying the pathomechanisms of AD. Nine AD-related lncRNAs were found in the dysregulated ceRNA network, and these lncRNA expressions were closely associated with neurodegenerative diseases such as AD and Parkinson's disease. Among them, five lncRNAs can be used as stable biomarkers for AD.

## Data Availability Statement

The datasets presented in this study can be found in online repositories. The names of the repository/repositories and accession number(s) can be found in the article/Supplementary Material.

## Ethics Statement

The studies involving human participants were reviewed and approved by the Neuroscience Center, Sir Run Run Shaw Hospital, School of Medicine, Zhejiang University. The patients/participants provided their written informed consent to participate in this study.

## Author Contributions

DX and CH conceived and supervised the research. SD analyzed the data. JX wrote the manuscript. JL and NL prepared figures and tables. DX identified the research and editorial manuscript. All authors read and approved the manuscript.

## Conflict of Interest

The authors declare that the research was conducted in the absence of any commercial or financial relationships that could be construed as a potential conflict of interest.
