# Supplementary figures and images for: A Signature of Five Long Non-Coding RNAs for Predicting the Prognosis of Alzheimer's Disease Based on Competing Endogenous RNA Networks
Source: Front Aging Neurosci. 2021 Jan 28;12:598606. doi: 10.3389/fnagi.2020.598606 (PMC7876075; doi:10.3389/fnagi.2020.598606)

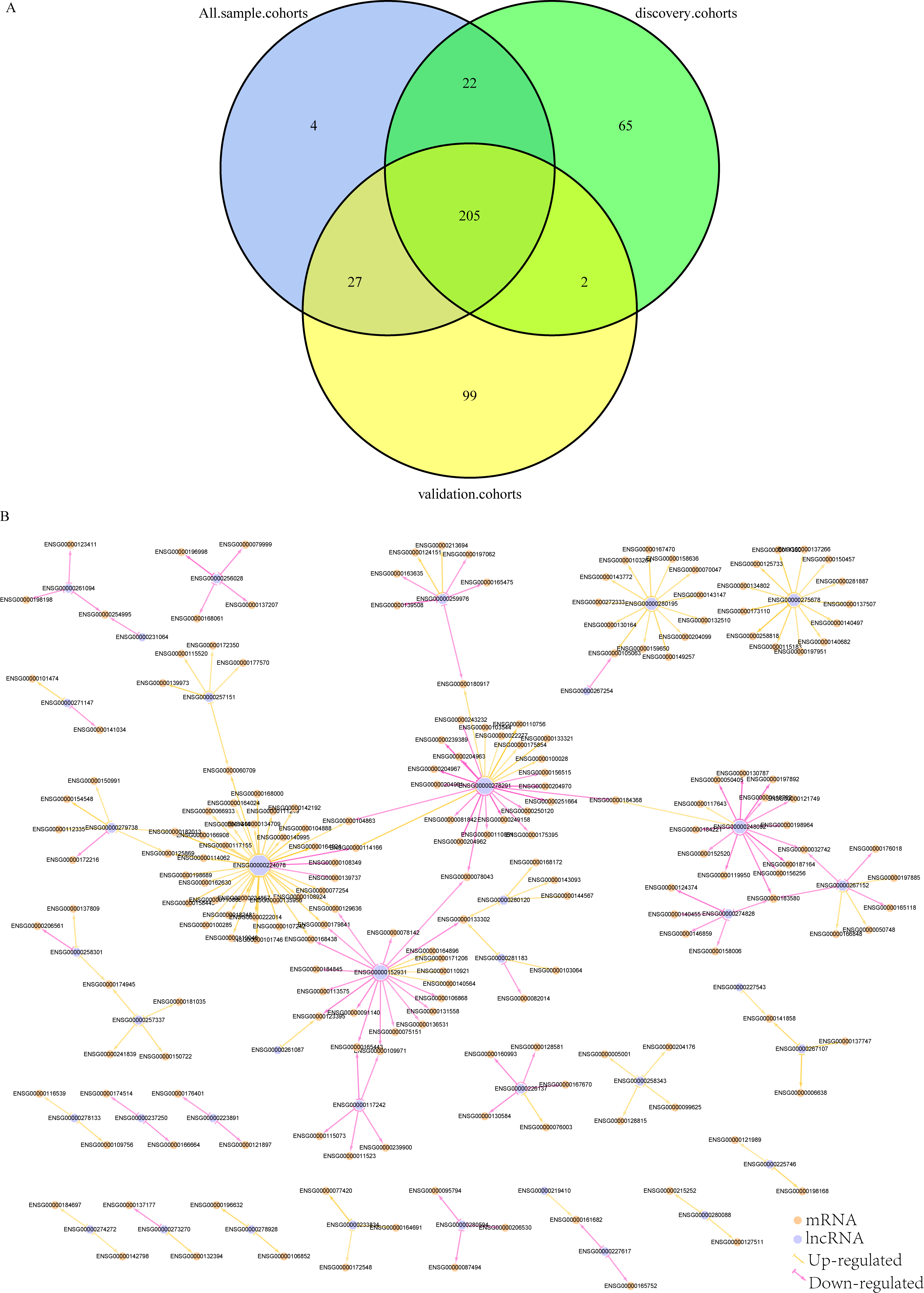

Supplement: Supplementary file 1 [file Image_1.TIF]

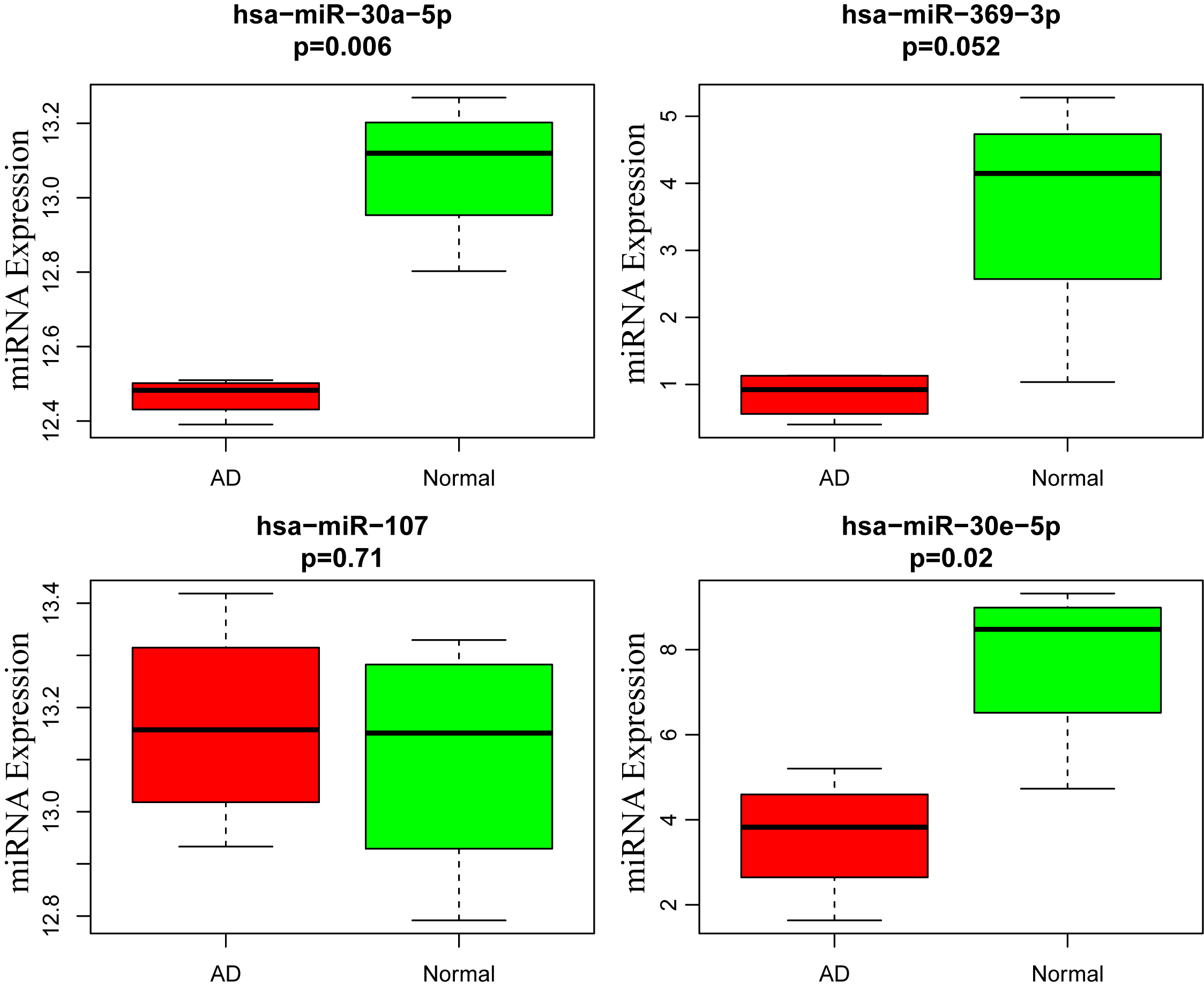

Supplement: Supplementary file 2 [file Image_2.TIF]

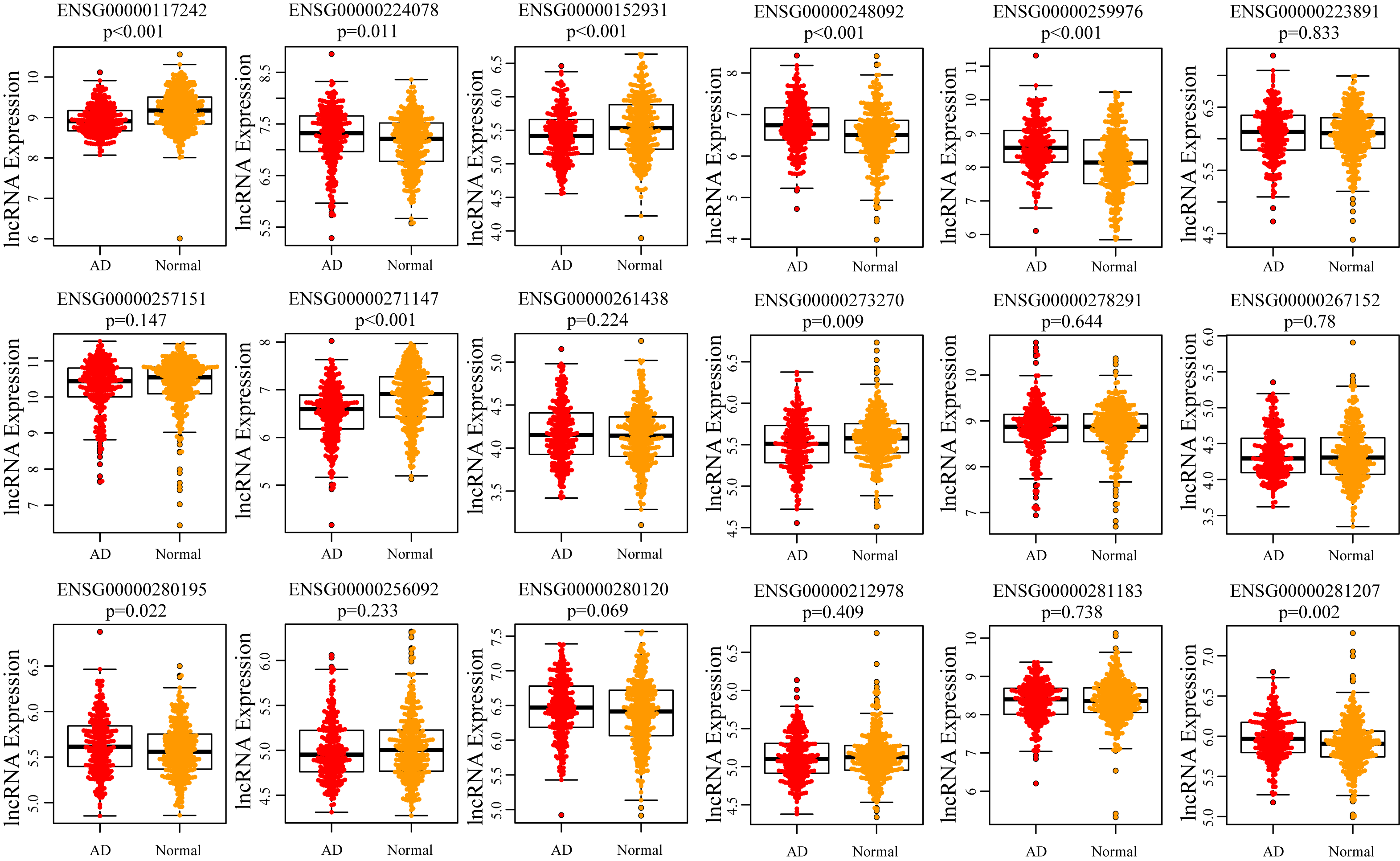

Supplement: Supplementary file 3 [file Image_3.TIF]

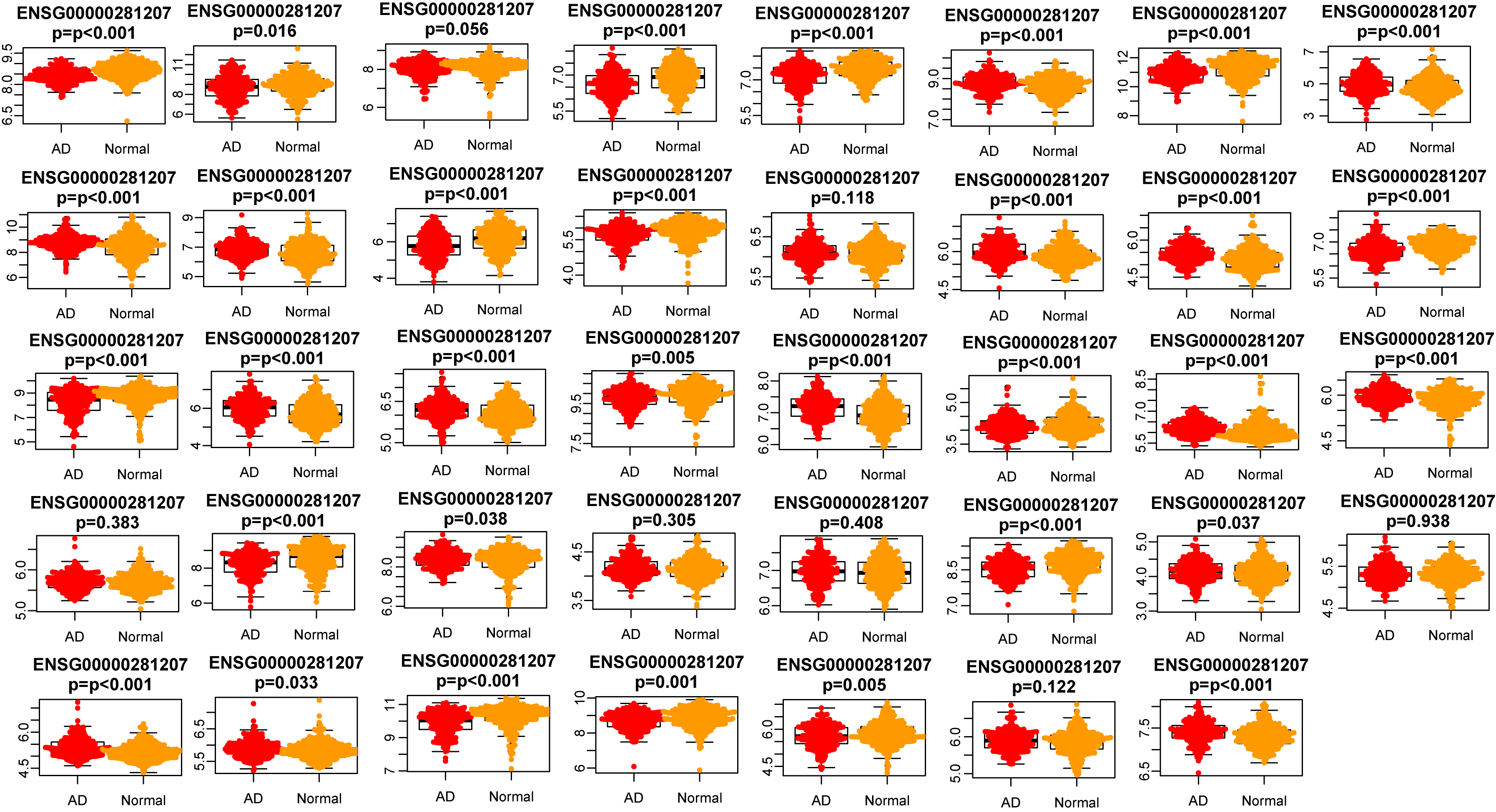

Supplement: Supplementary file 4 [file Image_4.TIF]
